# A 60-second interpretable voice model for early dementia screening

**DOI:** 10.1371/journal.pdig.0001552

**Published:** 2026-07-27

**Authors:** Kevin Mekulu, Faisal Aqlan, Hui Yang

**Affiliations:** 1 Harold and Inge Marcus Department of Industrial and Manufacturing Engineering, Pennsylvania State University, University Park, Pennsylvania, United States of America; 2 Department of Industrial Engineering, University of Louisville, Louisville, Kentucky, United States of America; University of California Los Angeles, UNITED STATES OF AMERICA

## Abstract

Early detection of cognitive impairment in assisted living is hindered by time-intensive tools like the Mini-Mental State Examination (MMSE) and the Montreal Cognitive Assessment (MoCA). We present a 60-second voice-based screening model that analyzes picture descriptions to estimate dementia risk. While recent deep learning approaches have shown promise on similar tasks, their lack of interpretability, large data requirements, and computational complexity limit clinical adoption. Using transcripts from the DementiaBank corpus, our model integrates traditional linguistic features (pause rate, pronoun use, syntactic complexity) with latent semantic dimensions extracted from language model embeddings. These semantic axes, interpretable constructs like “Drift & Hesitation” or “Over-detailed Narration”, consistently emerged as top predictors and may represent novel linguistic biomarkers of early decline. The final ElasticNet classifier is sparse, interpretable, and outperforms known non–deep learning baselines (AUC = 0.858), exceeding MMSE. Its simplicity enables deployment in mobile apps or in-room monitors, offering scalable, low-burden screening for early dementia. This work supports a shift toward linguistically grounded, tech-enabled cognitive care in aging populations.

## Author summary

Early-stage dementia often goes undetected in assisted living communities, where time constraints and staffing limitations make routine cognitive screening impractical. While standard tools like the MMSE and MoCA require trained administration and take 10–15 minutes, our research introduces a fast, interpretable alternative: a 60-second voice-based screening model using picture description tasks. We analyzed speech samples from individuals describing a common scene, extracting both traditional linguistic features (e.g., pauses, pronouns, sentence complexity) and deeper thematic patterns using modern language embeddings. Our model not only outperforms widely used tools in accuracy, but also reveals interpretable language patterns such as hesitation or over-description that may serve as early signs of cognitive decline. Lightweight and explainable, this approach is well suited for mobile apps or in-room monitors, enabling scalable, low-burden dementia screening in real-world care environments.

## Introduction

Cognitive impairment often goes undetected in assisted-living environments, where time constraints, staff shortages, and limited access to specialists hinder consistent screening. Yet early identification is critical, as Alzheimer’s disease progresses gradually over time, often before overt symptoms appear [1]. Widely used cognitive screening tools such as the Mini-Mental State Examination (MMSE) and the Montreal Cognitive Assessment (MoCA) are brief standardized tests designed to assess global cognitive function across domains including memory, attention, language, and visuospatial ability. While both instruments are clinically validated and widely used, they require trained administration and typically take 10–15 minutes per patient, limiting their scalability and frequency of use in real-world care settings [2,3].

Yet, language is among the first cognitive domains to show subtle changes in the earliest stages of dementia. Prior studies have demonstrated that features such as hesitation, reduced lexical diversity, and impaired syntactic structure emerge even before formal diagnoses [4,5]. Spontaneous speech, particularly in response to open-ended tasks like picture description, offers a natural, low-burden opportunity to capture these early markers.

Recent deep learning models have shown promise in identifying dementia from transcribed speech, but their lack of interpretability, large data requirements, and computational complexity remain barriers to clinical integration [6–9]. In contrast, we present a lightweight, interpretable model trained on 60-second responses to the Cookie Theft picture description task from the DementiaBank Pitt Corpus. Our model combines traditional hand-crafted linguistic features such as pause rate, pronoun ratio, and syntactic complexity with a set of latent semantic dimensions derived from language embeddings using singular value decomposition (SVD).

These semantic axes consistently ranked among the most predictive features and reveal interpretable discourse-level patterns that align with clinical intuition. For example, some axes highlight tendencies toward over-detailed narration or conceptual drift, behaviors often noted by clinicians but difficult to quantify. We propose that these latent dimensions represent a new class of candidate linguistic biomarkers for early cognitive decline.

## Materials and methods

### Ethics statement

All data used in this study were derived from a publicly available, IRB-approved dataset (DementiaBank). Participants provided informed consent prior to participation. No new data were collected, and all procedures followed ethical use and data protection guidelines established by the DementiaBank consortium.

### Dataset and participants

This study draws on the DementiaBank Pitt Corpus, a widely used benchmark for analyzing spontaneous speech in aging populations. Participants were asked to describe the standardized “Cookie Theft” picture, a well-known clinical image depicting a kitchen scene with multiple characters and simultaneous actions [10]. Although the task appears simple, just narrating what one sees, it is precisely this open-ended format that helps capture subtle linguistic markers of cognitive decline.

Our final dataset included 310 transcripts from 168 individuals diagnosed with Alzheimer’s Disease and 242 transcripts from 98 cognitively healthy controls. We excluded interviewer prompts and focused solely on participants’ speech to isolate natural linguistic production. Demographic details are presented in Table 1.

**Table 1:** Demographic characteristics of participants.

Type	Healthy Control (HC)	Alzheimer’s Disease (AD)
No. of Participants	98	168
Gender (M/F)	31 / 67	55 / 113
Age (mean ± SD)	64.7 ± 7.6	71.2 ± 8.4
Education (years)	14.0 ± 2.3	12.2 ± 2.6
MMSE Score	29.1 ± 1.1	19.9 ± 4.2

### Preprocessing

All transcripts were preprocessed using the spaCy library. Text was lowercased and lemmatized, and most punctuation was removed, except for ellipses and [pause] markers, indicators of disfluencies that can signal cognitive strain. This approach preserved clinically relevant cues while maintaining compatibility with downstream natural language processing.

### Feature extraction

To model participants’ speech patterns, we extracted two complementary layers of features.

#### Linguistic and domain features.

A total of 13 handcrafted linguistic features were computed, including word and sentence counts, average sentence length, type-token ratio (lexical richness), counts of various parts of speech (nouns, verbs, adjectives, adverbs), pronoun ratio, filler and pause rates, sentiment polarity, and idea density (clauses per sentence). These features mirror dimensions clinicians are trained to observe in narrative tasks [11].

#### Latent semantic features.

To capture deeper discourse themes, we used the all-MiniLM-L6-v2 transformer model to embed each transcript in a 384-dimensional space. The semantic axis labels (e.g., “Drift and Hesitation,” “Over-detailed Narration”) were assigned post hoc by the authors for interpretability, based on inspection of top-loading keywords and correlations with established linguistic features; no human annotation or manual labeling was used in model training. We then applied truncated singular value decomposition (SVD) [12] to reduce this space to 30 orthogonal axes. Each axis was interpreted by examining the top TF–IDF keywords and correlating the axes with known linguistic features, surfacing abstract constructs like hesitation, object focus, and over-description.

#### Feature matrix and scaling.

The final 43-dimensional feature matrix combined 13 handcrafted variables and 30 semantic axes. All features were standardized using z-scores prior to modeling.

### Model training and calibration

We trained a regularized logistic regression model using ElasticNet [13], which blends L1 and L2 penalties to promote sparsity and stability. Data were split into 80% training and 20% testing sets, with 5-fold cross-validation on the training set to optimize the ROC-AUC. To address class imbalance, we used balanced class weights. A range of ElasticNet mixing parameters ($\ell_{1}$ ratios = [0.1, 0.3, 0.5, 0.7, 0.9]) and regularization strengths (*C* = 10) were tested.

Because this model is intended for early screening, we calibrated the final classification threshold to achieve at least 90% sensitivity on the test set, prioritizing the detection of potential cases even at the cost of specificity. To mitigate class imbalance between Alzheimer’s disease and healthy control samples, balanced class weights were applied during model training.

### Interpretability analyses

Interpretability was a core design principle. Only 8 features received nonzero weights in the final model; three were handcrafted (pause rate, noun count, adverb count), while five were semantic axes (e.g., “Drift and Hesitation,” “Concise Scene-Setting”). These features are listed in Table 2, and visualized in Fig 2.

**Table 2:** Sparse feature coefficients and clinical descriptions.

Feature	Coefficient	Description
pause_rate	–0.30	Pause rate (per 100 words)
noun_count	+0.30	Total noun count
adv_count	–0.25	Total adverb count
SVD axis 0	+0.22	“Drift and Hesitation”
SVD axis 4	+0.18	“Concise Scene-Setting”
SVD axis 6	+0.15	“Uncertain Action”
SVD axis 12	+0.12	“Family and Object Focus”
SVD axis 15	–0.10	“Over-detailed Narration”

Each semantic axis was further interpreted using TF–IDF keyword analysis and correlations with linguistic variables. For example, Axis 0 (Drift and Hesitation) correlated with higher pronoun use and increased pause frequency. Axis 4 (Concise Scene-Setting) aligned with higher type-token ratio and lower pronoun use. These interpretations are summarized visually in Fig 3.

## Results

We evaluated the performance of our ElasticNet classifier on a held-out test set drawn from the DementiaBank Pitt Corpus. The model was trained using 80% of the available data and tested on the remaining 20%. Across all metrics, the system demonstrated strong screening performance, interpretability, and parsimony, making it a compelling candidate for real-world clinical deployment.

### Model performance

**Fig 1:**
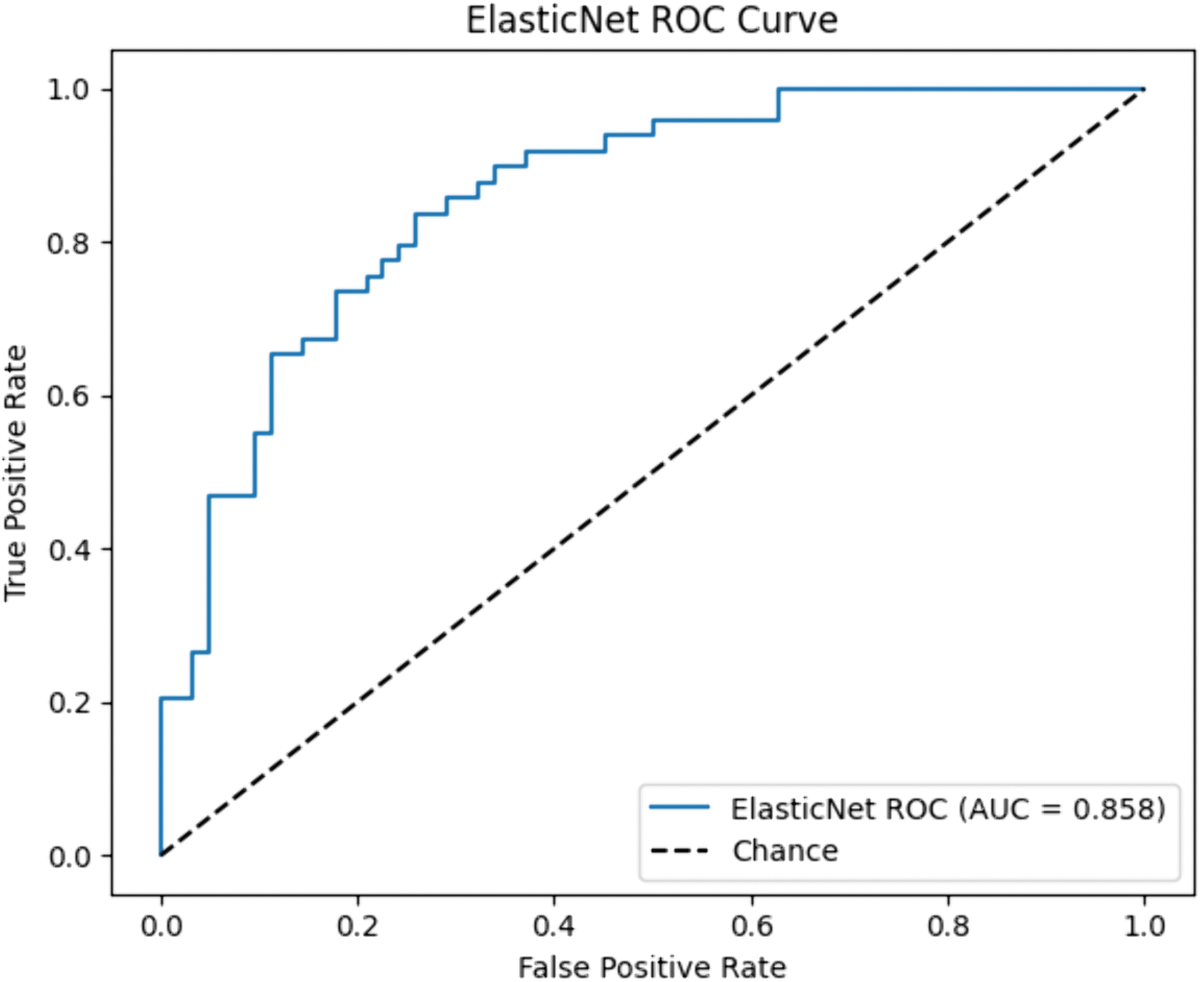
Receiver Operating Characteristic (ROC) curve for the ElasticNet model. AUC = 0.858 on the 20% hold-out set.

Our model achieved an area under the receiver operating characteristic (ROC) curve of 0.858 (Fig 1), outperforming traditional cognitive assessments like the MMSE (AUC ≈ 0.79) and MoCA (AUC ≈ 0.85). It also surpassed all previously reported non–deep learning baselines on the same picture description task, which typically score below 0.82 [14]. These results position our 60-second, speech-based system among the most accurate lightweight dementia screeners in the literature. In addition to comparisons with MMSE and MoCA, the proposed model achieves performance comparable to previously reported speech-based machine learning approaches evaluated on similar narrative speech tasks.

### Sensitivity and specificity

Given the model’s intended use in early screening, we calibrated its decision threshold to prioritize sensitivity. At a threshold of 0.37, the model identified 90% of dementia cases correctly, with a specificity of 65%. Even when lowering the threshold to 0.25, sensitivity remained above 90%, though specificity decreased to 55%. This trade-off is consistent with the clinical goal of minimizing false negatives, especially in settings where early intervention is critical.

### Model sparsity and feature contributions

**Fig 2:**
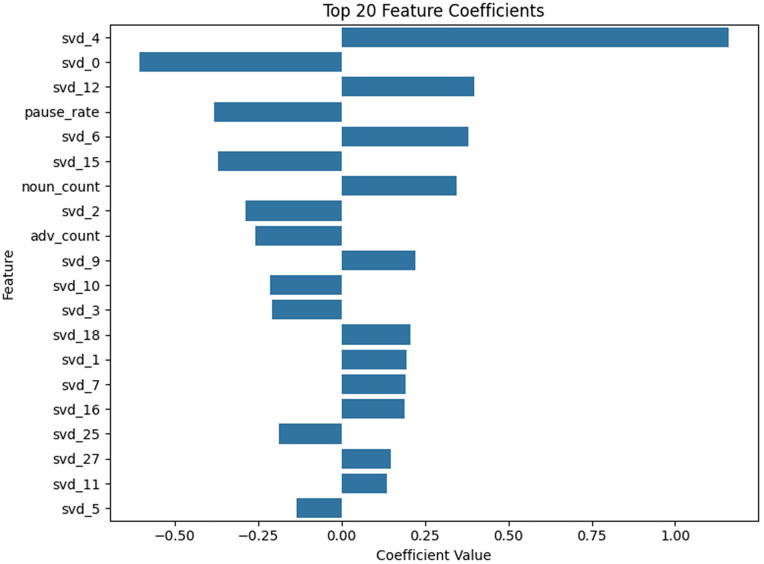
Top 20 feature coefficients from the ElasticNet model. Highlighted bars represent the 8 features with nonzero weights.

One of the strengths of the final classifier is its interpretability. Although the model was trained on 43 total features, 13 hand-crafted linguistic variables and 30 semantic dimensions derived via SVD; only 8 features received nonzero weights. These included pause rate (–0.30), noun count (+0.30), adverb count (–0.25), and five latent semantic axes. Fig 2 visualizes the top 20 coefficients, illustrating that a small and clinically interpretable subset of features accounts for the majority of predictive power.

### Semantic axes as linguistic biomarkers

Notably, several of the most predictive features were not explicitly designed by hand, but rather emerged from unsupervised semantic compression. By projecting sentence-level embeddings onto 30 orthogonal axes via truncated SVD, we identified discourse-level dimensions that offer unique insight into how dementia affects language.

Axis 0, labeled “Drift & Hesitation,” includes vague or uncertain language (e.g., “maybe”) and is positively correlated with both pronoun use (*r* = +0.34) and pause rate (*r* = +0.25). These patterns suggest lexical uncertainty and word retrieval difficulty. In contrast, Axis 4, “Concise Scene-Setting”, is characterized by grounded descriptive terms like “window” and “fall,” and aligns with higher lexical diversity and fewer pronouns, reflecting efficient narrative structure.

Axis 6, termed “Uncertain Action,” features verbs like “open” and “guess” and is tied to frequent pauses, indicating hesitation or degraded recall. Axis 12, “Family & Object Focus,” is anchored by concrete nouns such as “woman” and “dish,” and positively correlates with total noun usage, perhaps reflecting semantic anchoring. Finally, Axis 15, “Over-detailed Narration”, captures verbosity, sequencing modifiers and actions, and correlates with adverb count, suggesting disorganized or compensatory language strategies.

**Fig 3:**
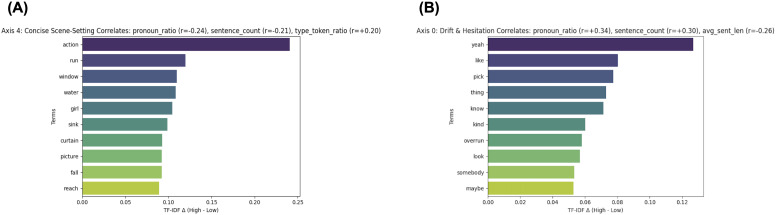
Keyword distributions and feature correlations for two interpretable semantic axes derived from SVD. **(A)** Axis 4: Concise Scene-Setting. **(B)** Axis 0: Drift & Hesitation.

Fig 3 presents two representative axes and their top keywords, illustrating how these latent dimensions capture distinct, clinically relevant aspects of narrative speech.

## Discussion

Detecting cognitive decline at an early stage, before symptoms become irreversible, remains a critical challenge in dementia care. In real-world environments such as assisted living facilities, routine cognitive screening is rarely performed due to staffing constraints, time limitations, and the logistical demands of traditional tools. Standard assessments like the MMSE or MoCA require trained administration and take several minutes per patient, limiting their scalability for frequent use. Our work introduces a more feasible alternative: a transparent voice-based model that assesses dementia risk using only 60 seconds of natural speech.

**Fig 4:**
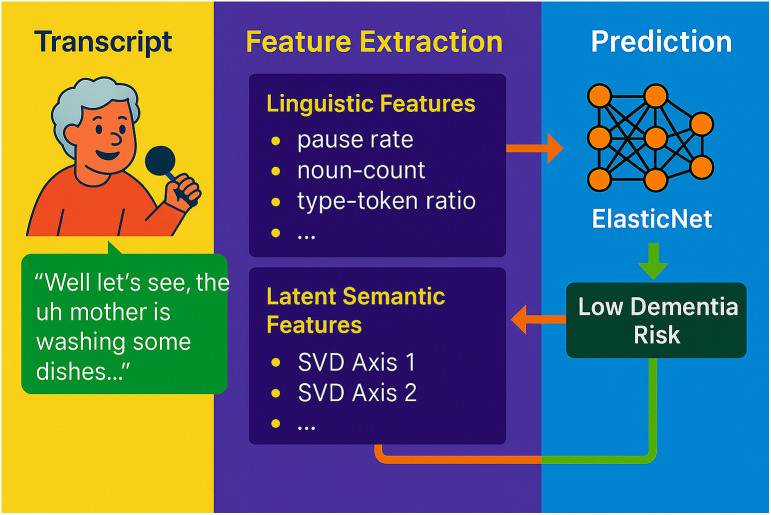
Overview of the dementia screening workflow. The system processes a 60-second picture description transcript, extracts both traditional linguistic features and latent semantic features, and uses an ElasticNet model to generate an interpretable dementia risk estimate. Figure created using ChatGPT(OpenAI).

To ensure interpretability and ease of deployment, we implemented a sparse ElasticNet logistic regression framework. The model achieves an AUC of 0.858 on held-out data, outperforming MMSE, MoCA and all known non–deep-learning baselines on the same task. Importantly, the model’s simplicity and transparency make it suitable for integration into mobile applications or in-room screening tools, enabling routine, scalable risk estimation in settings where early intervention is most critical. Rather than prioritizing black-box performance, our design emphasizes clinical relevance, interpretability, and practical deployment. The model achieves a strong AUC of 0.858 and relies on fewer than ten features, many of which are intuitive to clinicians. This performance compares favorably with prior non–deep-learning approaches on the same corpus: Fraser et al. [4] achieved 81% accuracy using 35 handcrafted linguistic features, and Orimaye et al. [14] reported AUC below 0.82. Notably, our lightweight model also approaches the performance of computationally intensive deep learning systems, such as the data2vec-based end-to-end model of Agbavor and Liang [15], which achieved AUC = 0.846 on a comparable Cookie Theft speech task, albeit without interpretability constraints. Traditional linguistic indicators such as pause rate, noun count, and adverb use are complemented by latent semantic axes extracted from sentence-level embeddings. These semantic dimensions capture patterns like conceptual drift, over-description, and uncertainty; they represent phenomena that clinicians often observe qualitatively but have lacked tools to measure consistently. We propose that such axes may serve as a new class of linguistically grounded biomarkers, offering novel insight into the subtle transformations in narrative structure that accompany early cognitive decline. The full workflow is illustrated in Fig 4.

**Fig 5:**
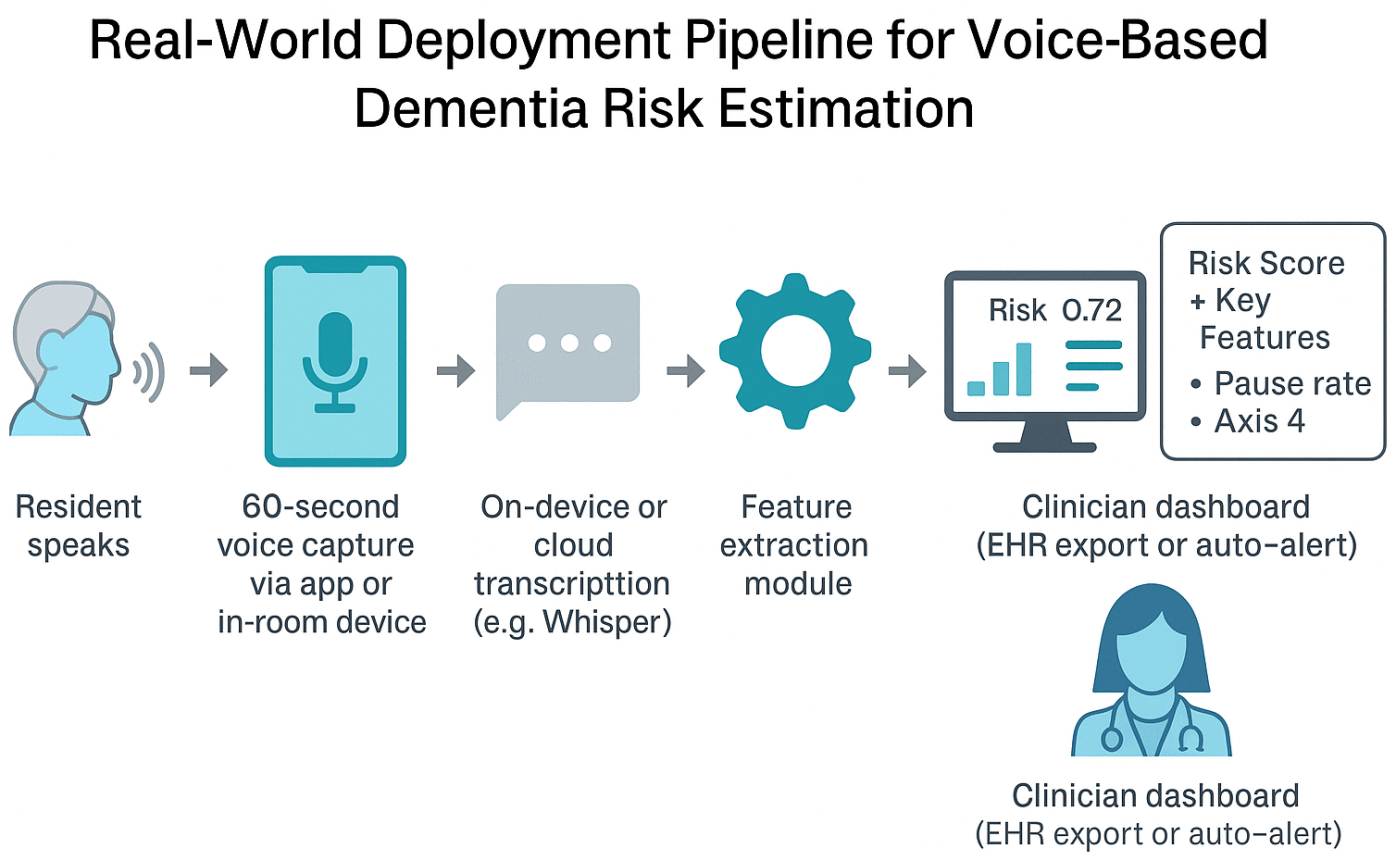
Real-world deployment pipeline for the voice-based dementia screening model. The system captures a 60-second speech sample, extracts linguistic and semantic features, and generates a transparent risk score to inform care staff. Designed for low-burden use in assisted living settings, it can be integrated into mobile applications or in-room monitors. Figure created using ChatGPT (OpenAI).

One of the most compelling aspects of this model is its ease of use. The proposed approach is designed to minimize burden relative to traditional cognitive assessments; however, practical deployment would still require appropriate hardware (e.g., a tablet or smartphone) and guided administration to ensure task comprehension and data quality. As illustrated in Fig 5, a short voice sample collected during a picture description task is sufficient for analysis. The system can be integrated into mobile applications or ambient in-room devices, providing an accessible and scalable method for continuous cognitive monitoring in environments where routine screening has traditionally been infeasible.

The model is calibrated to favor sensitivity; at a decision threshold of 0.37, it identifies 90 percent of dementia cases with 65 percent specificity. In early screening contexts, especially those involving high-risk populations, minimizing false negatives is clinically appropriate. Missed cases can delay intervention and increase long-term risk; false positives, by contrast, can be resolved through follow-up assessments.

### Limitations and future directions

Although promising, this work has limitations. Although the proposed model is motivated by early detection, the present dataset primarily reflects individuals with established Alzheimer’s disease, as indicated by mean MMSE scores. Validation in mild cognitive impairment (MCI) populations will be essential to assess sensitivity at earlier disease stages and is a key focus of ongoing and future work. The model was developed and evaluated on a single dataset, which, while well-established, may not fully represent the broader diversity of cognitive profiles, cultural backgrounds, or educational contexts. Repeated use of the same visual stimulus, the Cookie Theft image, may also introduce learning effects or cultural bias. To address this, we are developing a set of clinically vetted illustrations with varied narrative complexity and visual cues to support longitudinal use and broader generalizability.

Additionally, the model is not designed to be diagnostic; rather, it serves as an early screening tool that can flag individuals for further evaluation. Ongoing pilot studies in assisted living communities will help assess usability, effectiveness, and staff adoption in real-world settings. Given the high co-occurrence of cognitive decline and depression, future iterations of this system will include a parallel depression screening module, creating a more comprehensive voice-based mental health tool for aging populations.

Because this study relies on transcript-based inputs, future work will be required to assess robustness under real-world acoustic conditions, including background noise and automated speech recognition errors.

Taken together, these results demonstrate the potential of voice as a scalable digital biomarker. A single minute of speech may be enough to detect meaningful changes in cognition; with continued validation and clinical integration, this approach may offer a path toward more proactive, personalized care.

### Real-world deployment

Fig 5 summarizes the envisioned pipeline for deploying the model in real-world clinical or residential care settings, beginning with a 60-second voice sample collected via a tablet or smartphone, followed by automated on-device transcription, feature extraction, and risk prediction. The resulting dementia risk score, along with the most influential features, can then be displayed on a clinician dashboard to support early decision-making and patient monitoring.

## Conclusion

This study presents a fast, interpretable model for dementia screening based entirely on natural language. Using a brief picture description task, we extract both handcrafted linguistic features and latent semantic dimensions from sentence embeddings. The resulting classifier achieves strong predictive performance (AUC = 0.858) with only eight active features, outperforming traditional tools such as MMSE and MoCA on the same task.

Beyond classification, our approach offers transparency and thematic insight: semantic axes such as “Drift and Hesitation” or “Over-detailed Narration” provide interpretable links between language and cognition. The system is lightweight, low-burden, and suitable for deployment in mobile apps or in-room monitors, allowing for routine screening and potential longitudinal monitoring. As dementia care increasingly shifts toward early intervention, models like ours may help bridge the gap between unnoticed cognitive changes and timely clinical action.
